# Phase 1 open-label study of panobinostat, lenalidomide, bortezomib + dexamethasone in relapsed and relapsed/refractory multiple myeloma

**DOI:** 10.1038/s41408-021-00407-5

**Published:** 2021-02-05

**Authors:** Jacob P. Laubach, Sascha A. Tuchman, Jacalyn M. Rosenblatt, Constantine S. Mitsiades, Kathleen Colson, Kelly Masone, Diane Warren, Robert A. Redd, Dena Grayson, Paul G. Richardson

**Affiliations:** 1 Dana-Farber Cancer Institute, Harvard Medical School, Boston, MA USA; 2 University of North Carolina, Chapel Hill, NC USA; 3 Beth Israel Deaconess Medical Center, Harvard Medical School, Boston, MA USA; 4 Dana-Farber Cancer Institute, Department of Data Sciences, Boston, MA USA; 5 Secura Bio, Inc, San Diego, CA USA

**Keywords:** Myeloma, Combination drug therapy

## Abstract

Additional therapeutic options are needed for relapsed and refractory multiple myeloma (RRMM). We present data from a phase 1b, open-label, dose-escalation study (NCT01965353) of 20 patients with RRMM (median age: 63 years [range: 50–77]) and a median of four prior regimens (range: 2–14); 85% had refractory disease (lenalidomide [80%]; bortezomib [75%]; lenalidomide and bortezomib [50%]). Patients received a median of six cycles (range: 1–74) of panobinostat (10 or 15 mg), lenalidomide 15 mg, bortezomib 1 mg/m^2^, and dexamethasone 20 mg (pano-RVd). Median follow-up was ~14 months. Six dose-limiting toxicities were reported (mostly hematological); maximum tolerated dose of panobinostat (primary endpoint) was 10 mg. Most common adverse events (AEs) were diarrhea (60%) and peripheral neuropathy (60%); all grade 1/2. Grade 3/4 AEs occurred in 80% of patients and included decreased neutrophil (45%), platelet (25%) and white blood cell (25%) counts, anemia (25%) and hypophosphatemia (25%). No treatment-related discontinuations or mortality occurred. In evaluable patients (*n* = 18), overall response rate was 44%, and clinical benefit rate was 61%. Median duration of response was 9.2 months; progression-free survival was 7.4 months; overall survival was not reached. Pano-RVd proved generally well-tolerated and demonstrated potential to overcome lenalidomide and/or bortezomib resistance.

## Introduction

Treatment of multiple myeloma (MM), an incurable plasma-cell neoplasm, has changed substantially in recent decades^1^. Rapidly evolving treatment standards have led to improvements in overall survival (OS), as well as the depth and duration of response (DOR)^2–4^. Nevertheless, most patients ultimately relapse and require subsequent lines of therapy, with the depth of response and DOR to each successive regimen typically decreasing over time^1^. However, different emerging classes of agents, which can be combined in triplet or even quadruplet regimens, have provided clinicians with several new therapeutic options for relapsed and refractory MM (RRMM) patients.

Histone deacetylase inhibitors (HDACis), which influence transcriptional activation and other nuclear events by increasing histone acetylation, have been developed in recent years as a treatment for RRMM^1,5–7^. Histone deacetylases (HDACs) are overexpressed in MM, leading to reduced expression of tumor suppressor genes and, consequently, increased growth and proliferation of tumor cells. Unsurprisingly, overexpression of class I HDACs is associated with a poorer prognosis in MM^8^.

Broad-spectrum HDACis can enhance the anti-MM activity of proteasome inhibitors through multiple non-mutually exclusive mechanisms, including the transcriptional repression of ubiquitin/proteasome pathway genes and drivers of tumor cell survival and treatment resistance^9,10^, as well as the inhibition of the aggresome, which is an alternative route for protein degradation^11^. Panobinostat is among the most potent HDACis in clinical development and the only HDACi approved for the treatment of RRMM, in combination with bortezomib and dexamethasone^12,13^. In the phase 2 PANORAMA 2 trial, heavily pre-treated, bortezomib-refractory patients with RRMM had an overall response rate (ORR) of 34.5% with panobinostat plus bortezomib and dexamethasone (pano-Vd), highlighting that the addition of panobinostat can overcome resistance to prior therapeutic agents, including bortezomib^14^. In the randomized phase 3 PANORAMA 1 study, pano-Vd significantly improved median progression-free survival (PFS) by 12.5 vs. 4.7 months (hazard ratio 0.47; 95% confidence interval [CI] 0.31, 0.72) in patients who had received ≥2 prior regimens including bortezomib and an immunomodulatory drug (IMiD), and nearly tripled the rate of complete response/near complete response, compared with placebo-Vd (22% vs. 8% for pano-Vd and placebo-Vd, respectively)^5,6,15^.

The optimal dose and schedule of pano-Vd was confirmed in the randomized phase 3 PANORAMA 3 trial as 20 mg thrice weekly; DOR with this regimen was 22.6 months and tolerability was improved with subcutaneous administration of bortezomib with only 11.5% of patients in the 20 mg TIW dosing group reporting grade 3/4 diarrhea compared with intravenous delivery of bortezomib, as was standard practice at the time of previous clinical trials.^16^

Broad-spectrum HDACis also enhance the anti-MM activity of IMiDs, such as lenalidomide, by suppressing diverse oncogenic transcriptional programs^10^, including the interferon regulatory factor-4/MYC axis^17^. In a phase 2 study of patients with RRMM, panobinostat plus lenalidomide and dexamethasone (pano-Rd) demonstrated an encouraging ORR (41%) and median PFS (7.1 months) in patients with high-risk, lenalidomide-refractory (81%), and/or bortezomib-refractory (52%) MM, suggesting that panobinostat is also able to overcome resistance to IMiDs.

These data provided the rationale for investigating the quadruplet regimen, panobinostat, lenalidomide, bortezomib and dexamethasone (pano-RVd), in heavily pre-treated patients, particularly in those refractory to proteasome inhibitors and/or IMiDs for whom additional therapy options are needed. Here, we report the findings of a phase 1b dose-escalation study of pano-RVd with extended follow-up.

## Methods

### Study design and objectives

This study was an open-label, multicenter, phase 1b, study of pano-RVd in RRMM (NCT01965353). Primary objectives were to identify the maximum tolerated dose (MTD) of panobinostat in combination with RVd and to evaluate the safety profile of pano-RVd. Secondary objectives were to evaluate ORR, DOR, time to progression (TTP), PFS and OS. A modified Fibonacci design was used, with 3–6 patients planned at each dose level followed by a dose-expansion phase with an additional ten patients to evaluate the tolerability of the MTD.

The study was conducted in accordance with the Declaration of Helsinki, US Code of Federal Regulations governing clinical study conduct, state laws and Dana-Farber/Harvard Cancer Center research policies and procedures. The institutional review board for each center approved the protocol and amendments. All patients provided written informed consent before enrollment.

### Patient eligibility

Eligible patients were aged ≥18 years with measurable RRMM (2011 International Myeloma Working Group consensus)^18^, an Eastern Cooperative Oncology Group performance status <2, and had received ≥2 lines of therapy. Patients with primary refractory disease, prior HDACi treatment, creatinine clearance <45 mL/min, platelet count <75,000 cells/mm^3^, absolute neutrophil count <1500 cells/mm^3^, or hemoglobin level <8.0 g/dl at screening were excluded. Patients with ≥grade (G) 2 peripheral neuropathy (PN) or hepatic impairment (bilirubin > 1.5 × institutional upper limit of normal, or aspartate aminotransferase, alanine aminotransferase, or alkaline phosphatase >2 × institutional upper limit of normal) within 21 days of study therapy initiation were also excluded.

### Treatments administered

**Fig. 1:**
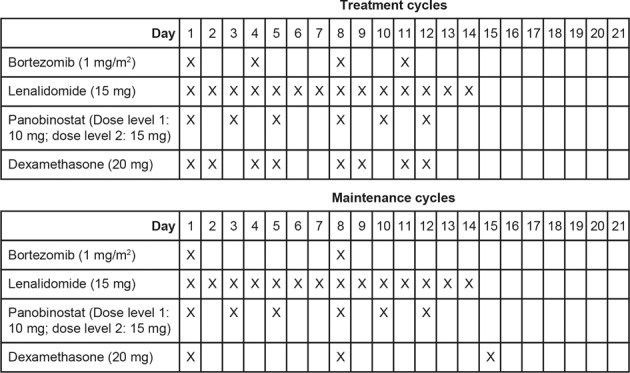
Each cycle consisted of 21 days, patients received eight treatment cycles before switching to maintenance cycles. Progression to the next higher dose level occurred, if appropriate, when the safety and tolerability of the prior dose level(s) had been determined at the end of the first cycle.

Patients received oral panobinostat 10 mg or 15 mg plus subcutaneous bortezomib 1 mg/m^2^, oral lenalidomide 15 mg, and oral dexamethasone 20 mg in 21-day cycles per the schema in Fig. 1. After eight cycles, patients switched to a maintenance schedule, with reduced bortezomib and dexamethasone dosing. Treatment continued until disease progression, unacceptable toxicity, consent was withdrawn, or discontinuation was in the best interest of the patient. Doses could be held for up to 21 days or reduced to manage therapy-related adverse events (TRAEs).

Necessary concomitant medications were allowed, except for those which may cause QTcF prolongation or induce torsades de pointes. Leukocyte growth factors were only administered during cycle 1 in the event of a dose-limiting toxicity (DLT) (if appropriate), but could be prescribed for severe neutropenia after cycle 1.

### Dose escalation, MTD, and DLTs

A standard 3 + 3 dose-escalation schema was used (Supplementary Fig. S1). If ≥2 of 3 patients within a cohort experienced a DLT during the dose-escalation phase, the dose immediately below the current dose was defined as the MTD. The 3 + 3 schema was chosen, rather than an alternative methodologic phase 1 study design, due to the limited number of patients in the study and the fact that only three dose levels were planned. DLTs were assessed during the first cycle and defined as a QTcF interval >500 ms, or an absolute increase of >60 ms, a ≥ G3 non-hematological AE or a G4 hematological AE (including thrombocytopenia with platelets <25,000/mm^3^ on >1 occasion, or G4 neutropenia for >5 days and/or resulting in neutropenic fever on two occasions). Lymphopenia, an AE associated with bortezomib use, was not considered a DLT. Inability to take ≥75% of the planned study drug doses, or receive day 1 doses for cycle 2 due to a drug-related AE occurring in cycle 1, were also considered DLTs.

### Assessments

AEs, graded using Cancer Therapy Evaluation Program Version 4 of the National Cancer Institute Common Terminology Criteria for Adverse Events^19^, were assessed throughout the study and for 30 days after completion of study therapy. Patients who discontinued for any reason other than disease progression were followed every three months until disease progression. Disease response/progression were assessed at the start of each treatment and maintenance cycle, at the end of study, and during the follow-up phase. Disease response was assessed locally using the International Myeloma Working Group Response Criteria^18^ with M-protein quantification and immunofixation in serum and 24-h urine samples, and serum-free light chain testing. OS was assessed every three months after disease progression.

### Statistical analyses

Data cut-off was 24 January 2019. Descriptive statistics are provided for the reported outcomes. All participants who had received ≥1 dose of any study treatment were evaluated for AEs from treatment initiation. All patients who had received study treatment and had ≥1 follow-up assessment were included in the response evaluation. Only patients who responded to treatment (≥minimal response) were included in the DOR analysis. DOR, TTP, PFS, and OS were estimated using Kaplan–Meier methodology. DOR was measured as the time from initiation of first response to time of disease progression, death, or last follow-up (for those patients who had not progressed or died). TTP was defined as time from registration to progression or to last follow-up (for those who had not progressed). PFS was defined as the time from registration to disease progression, death, or last follow-up (for those who had not progressed or died). OS was defined as time from registration to death or last follow-up (for patients who had not died).

## Results

### Patient characteristics

Between November 2013 and October 2016, 20 patients were enrolled: median age was 63 years (range: 50–77), 70% male (Table 1). Cytogenetic data were available for 17 patients; two had t(4;14), and one had t(14;16). The median number of prior lines of treatment was four (range: 2–14); five patients had received >5 prior lines of treatment. Overall, 95% and 90% had been previously treated with bortezomib and lenalidomide, respectively. Most patients (*n* = 17, 85%) had refractory disease (lenalidomide [80%]; bortezomib [75%]; lenalidomide and bortezomib [50%]; dexamethasone [85%]).

**Table 1:** Patient baseline characteristics.

	Total population *n* = 20 (%)
Male	14 (70)
Race	
Black/African American	4 (20)
White	15 (75)
Other	1 (5)
Age, median (range)	63 (50–77)
Age group	
≤55	4 (20)
56–65	9 (45)
>65	7 (35)
Disease status	
Relapsed	3 (15)
R/R	17 (85)
ECOG PS	
0	9 (45)
1	11 (55)
ISS classification	
I	5 (25)
II	7 (35)
III	7 (35)
Missing	1 (5)
Number of prior treatments, median (range)	4 (2–14)
Prior treatment	
Bortezomib	19 (95)
Dexamethasone	20 (100)
Lenalidomide	18 (90)
Pomalidomide	15 (75)
Carfilzomib	0
Refractory to any prior treatment	
Bortezomib	15 (75)
Dexamethasone	17 (85)
Lenalidomide	16 (80)
Refractory most recent prior treatment	
Bortezomib	9 (45)
Dexamethasone	16 (80)
Lenalidomide	2 (10)
Prior autologous transplants	
1	9 (45)
Cytogenetics	
No FISH failure	17 (85)
*t* (4;14)^a^	2 (10)
*t* (14;16)^b^	1 (5)

*ECOG PS* Eastern Cooperative Oncology Group Performance Status, *FISH* fluorescence in situ hybridization, *ISS* International Staging System, *R/R* relapsed/refractory.

^a^Missing: *n* = 9.

^b^Missing: *n* = 10.

### MTD and DLTs

Three patients were treated with panobinostat 10 mg; one experienced a DLT; per protocol, three additional patients were treated with panobinostat 10 mg. No further DLTs were reported, and the dose was escalated. Three patients were treated with panobinostat 15 mg; two experienced DLTs. One of these DLTs (syncope) was a pre-existing condition and not considered related to study treatment, thus, one additional patient was treated with panobinostat 15 mg; this patient subsequently experienced a DLT (thrombocytopenia). Therefore, 10 mg was defined as the MTD for panobinostat in the pano-RVd combination. In the expansion phase, ten patients were treated with pano-RVd with panobinostat dosed at 10 mg; two experienced a DLT. Overall, four patients experienced one DLT, one patient experienced two DLTs and one patient experienced three DLTs (Table 2).

**Table 2:** Dose limiting toxicities.

	*n* (panobinostat dose)
Decreased platelet count	3 (15 mg, *n* = 2; 10 mg, *n* = 1)
Decreased neutrophil count	2 (15 mg)
Fatigue	1 (15 mg)
Hyperglycemia	1 (10 mg)
Hypophosphatemia	1 (10 mg)
Syncope	1^a^ (15 mg)

^a^Not related to treatment.

### Treatment summary

Patients completed a median of six cycles (range: 1–74) (Supplementary Table S1). Three patients completed one cycle before withdrawing due to progressive disease. Eight patients completed all eight treatment cycles and ≥1 cycle of maintenance therapy. At the data cut-off date, one patient remained on treatment (Supplementary Fig. S2).

### Safety and tolerability

All 20 patients experienced ≥1 TRAE. The distribution of worst overall TRAEs experienced was G1 (*n* = 1), G2 (*n* = 3), G3 (*n* = 11) or G4 (*n* = 5). The most common TRAEs included diarrhea (60%, all were G1/2), PN (60%), anemia (55%), fatigue (55%), neutropenia (55%), constipation (50%) and hypokalemia (50%) (Table 3). The median (95% CI) duration of neutropenia and thrombocytopenia AEs was 7 (4; 11) days and 8 (4; 11) days, respectively. Overall, 37 G3/4 TRAEs were reported; the most common were decreased neutrophil (45%), platelet (25%) and white blood cell (25%) counts; anemia (25%); and hypophosphatemia (25%). The rate of cardiac TRAEs was low (atrial fibrillation, *n* = 1; atrial flutter, *n* = 1; sinus bradycardia, *n* = 1; sinus tachycardia, *n* = 1; palpitations, *n* = 1; prolongation of QTcF interval, n = 1; heart racing, *n* = 1; chronic right bundle branch block, *n* = 1; ectopy and murmur *n* = 1), and all events were G1/2. No G3/4 diarrhea TRAEs were observed. The only reported G4 TRAEs were decreased platelet (25%) and white blood cell counts (5%). In total, nine serious AEs were experienced by six patients, including treatment-related hyperglycemia, decreased neutrophil count, fatigue, and decreased platelet count. TRAE rates were generally balanced in patients in patients <65 (*n* = 11) and ≥65 (*n* = 9) years old, although G4 thrombocytopenia TRAEs were more common in patients ≥65 years old.

**Table 3:** AEs experienced by ≥20% of patients and all grade 4 AEs.

	Total, *n* (%)	Grade 1	Grade 2	Grade 3	Grade 4
	(*n* = 20)	*n* (%)	*n* (%)	*n* (%)	*n* (%)
Blood and lymphatic system disorders					
Anemia	11 (55)	2 (10)	4 (20)	5 (25)	–
Neutrophil count decreased	11 (55)	1 (5)	1 (5)	9 (45)	–
Platelet count decreased	9 (45)	4 (20)	–	–	5 (25)
White blood cell count decreased	7 (35)	–	2 (10)	4 (20)	1 (5)
Blood and lymphatic system disorders – other	4 (20)	1 (5)	3 (15)	–	–
Bruising	4 (20)	4 (20)	–	–	–
Cardiac disorders					
Dizziness	5 (25)	4 (20)	1 (5)	–	–
Cardiac disorders – other	4 (20)	4 (20)	–	–	–
Eye disorders					
Eye disorders—other	4 (20)	2 (10)	2 (10)	–	–
Gastrointestinal disorders					
Diarrhea	12 (60)	7 (35)	5 (25)	–	–
Constipation	10 (50)	6 (30)	4 (20)	–	–
Nausea	7 (35)	5 (25)	2 (10)	–	–
Gastrointestinal disorders—other	5 (25)	3 (15)	2 (10)	–	–
Dysgeusia	4 (20)	2 (10)	2 (10)	–	–
General disorders and administration site conditions					
Fatigue	11 (55)	1 (5)	9 (45)	1 (5)	–
Edema limbs	7 (35)	4 (20)	3 (15)	–	–
Infections and infestations					
Upper respiratory infection	8 (40)	–	8 (40)	–	–
Infections and infestations – other	6 (30)	2 (10)	2 (10)	2 (10)	–
Investigations					
Hypokalemia	10 (50)	8 (40)	2 (10)	–	–
Hypomagnesemia	8 (40)	8 (40)	–	–	–
Hypophosphatemia	7 (35)	1 (5)	1 (5)	5 (25)	–
Hypocalcemia	5 (25)	4 (20)	1 (5)	–	–
Hyponatremia	5 (25)	5 (25)	–	–	–
Metabolism and nutrition disorders					
Anorexia	7 (35)	5 (25)	2 (10)	–	–
Hyperglycemia	7 (35)	4 (20)	2 (10)	1 (5)	–
Musculoskeletal and connective tissue disorders					
Musculoskeletal and connective tissue disorder – other	4 (20)	2 (10)	2 (10)	–	–
Nervous system disorders					
Peripheral sensory neuropathy	12 (60)	6 (30)	6 (30)	–	–
Psychiatric disorders					
Insomnia	6 (30)	3 (15)	3 (15)	–	–
Respiratory, thoracic, and mediastinal disorders					
Cough	6 (30)	1 (5)	5 (25)	–	–
Dyspnea	6 (30)	3 (15)	2 (10)	1 (5)	–

AEs that were possibly, probably, or definitely related to any study treatments are reported. Each AE represents the highest grade for that AE per patient, so patients may be included in each row at most once.

*AE* adverse event.

There were no instances of treatment-related death or study discontinuation. Dose levels of lenalidomide, bortezomib, dexamethasone, and panobinostat were reduced in at least one cycle for three, six, six, and three patients, respectively. Dose reductions for panobinostat and lenalidomide only occurred in patients in the panobinostat 15 mg cohort. At least one cycle of the pano-RVd regimen was delayed in 18 patients. The most common reason for dose holds was AEs (73%), of which upper respiratory infection, decreased neutrophil count and peripheral sensory neuropathy were the most common (Supplementary Table S2). Reasons for treatment withdrawal included progressive disease (85%), death (5%) and autologous stem cell transplantation (5%).

### Responses and outcomes

**Fig. 2:**
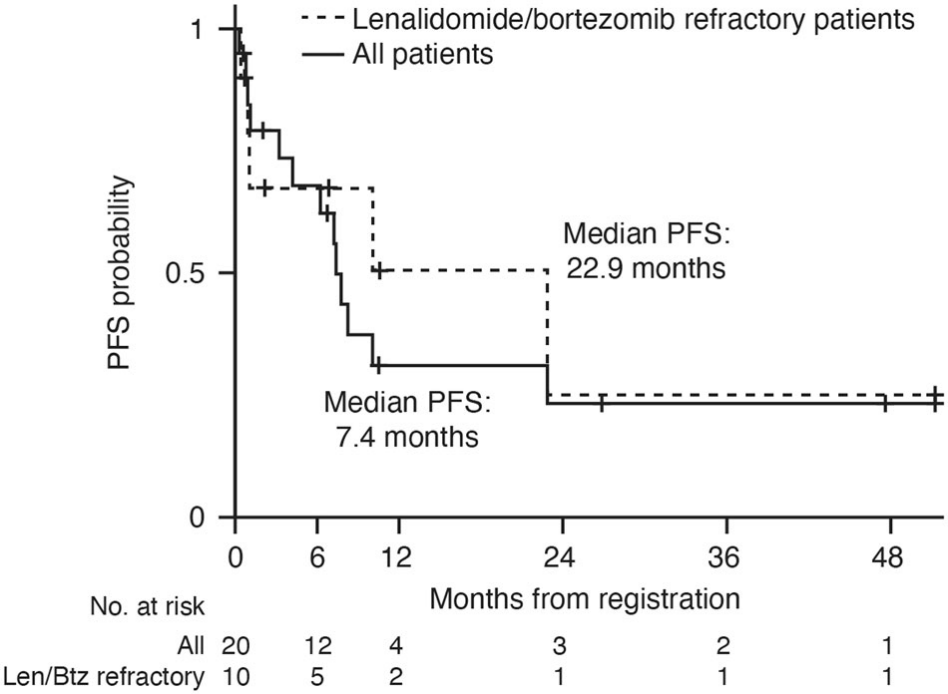
Kaplan–Meier distributions of PFS in all 20 patients receiving pano-RVd and lenalidomide/bortezomib-refractory patients. *Len/Btz* lenalidomide/bortezomib. *Pano-RVd* panobinostat/lenalidomide/bortezomib/dexamethasone, *PFS* progression free survival.

The median follow-up was ~14 months. Per protocol, two patients were not eligible for response evaluation; one died due to factors unrelated to study treatment 2 weeks after starting study treatment, and one withdrew from the study 3 days after starting treatment, due to rapid disease progression. In response-evaluable patients (*n* = 18), the rate of very good partial response or better was 17%, the ORR (≥partial response) was 44% (90% CI 24, 66) and the clinical benefit response rate (≥minimal response) was 61% (90% CI 39, 80) (Table 4). In patients refractory to both lenalidomide and bortezomib (*n* = 10), the ORR was 30% (90% CI 9, 61) and the clinical benefit rate was 50% (90% CI 22, 78). Median time-to-first response was approximately 1 month (range: 0.79–4.6), and the median DOR was 9.2 months (range: 2.1–50.4 months). At data cut-off, four patients had a DOR of at least 10 months, median (95% CI) TTP was 7.8 (7.2, not reached) months, median (95% CI) PFS was 7.4 (4.2, not reached) months (Fig. 2), and median (95% CI) OS had not been reached (13.5, not reached). In patients refractory to both lenalidomide and bortezomib, median (95% CI) DOR was 22.1 (9.2, not reached) months, median (95% CI) TTP and PFS were both 22.9 (0.9, not reached) months (Fig. 2), and median (95% CI) OS was not reached (6.8, not reached).

**Table 4:** Patient responses.

All patients, *N* = 18^a^	*N* (%)	90% CI
Stringent complete response	1 (6)	NA
Complete response	0 (0)	NA
Very good partial response	2 (11)	NA
Partial response	5 (28)	NA
Minimal response	3 (17)	NA
Stable disease	7 (39)	NA
Overall response rate (partial response or better)	8 (44)	24, 66
Clinical benefit rate (minimal response or better)	11 (61)	39, 80
Patients refractory to both lenalidomide and bortezomib, *N* = 10	*N* (%)	90% CI
Overall response rate (partial response or better)	3 (30)	9, 61
Clinical benefit rate (minimal response or better)	5 (50)	22, 78

*CI* confidence interval, *NA* not applicable. M-spike evaluation.

^a^Non evaluable: *n* = 2 (10%).

## Discussion

Despite recent approvals of new therapeutic agents for RRMM, nearly all patients ultimately still experience disease relapse. The pano-RVd regimen investigated in this study provides a potential treatment option that can restore responses in heavily treated patients with refractory disease. The MTD of panobinostat in the pano-RVd regimen was 10 mg, dosed in a 2-weeks-on/1-week-off schedule, in combination with bortezomib 1 mg/m^2^, lenalidomide 15 mg and dexamethasone 20 mg. Although all patients experienced at least one TRAE, no patients discontinued therapy or were withdrawn from the study due to a TRAE. Importantly, this regimen demonstrated promising activity in RRMM patients, including those who were refractory to bortezomib and/or lenalidomide and had received a median of four prior lines of therapy. Furthermore, DOR, TTP, and PFS efficacy outcomes were favorable, even in patients refractory to both lenalidomide and bortezomib.

Most patients in the study were refractory to bortezomib or lenalidomide (75% and 80%, respectively) and 50% were refractory to both bortezomib and lenalidomide. The overall ORR of 44% and clinical benefit rate of 61% demonstrates that pano-RVd can provide disease control in patients previously treated with and resistant to lenalidomide and/or bortezomib, a finding that is consistent with previous studies involving panobinostat-containing regimens^7,14^. This observation is likely due to the unique epigenetic mechanism of action of panobinostat, which targets multiple pathways that contribute to high-risk biology in MM and abrogates resistance to more established agents caused by epigenetic changes^9,10,20^.

AEs commonly associated with regimens incorporating panobinostat, bortezomib, and/or lenalidomide include neutropenia, diarrhea, PN, and thrombocytopenia^2,13,21^. The most commonly reported TRAEs in this study included diarrhea and PN, which were all G1/2. This finding contrasts with the phase 3 PANORAMA 1 study in which 25% of patients experienced G3/4 diarrhea and 18% experienced G3/4 PN^5,6^. The toxicity profile differences observed between the present study and PANORAMA 1 may be related to the different method of bortezomib administration (subcutaneous vs. intravenous) and the reduced dose of panobinostat (10–15 mg vs. 20 mg). At the MTD for pano-RVd, G3/4 AEs were experienced by 80% of patients, the majority of which were hematological. This high percentage of G3/4 AEs reflects chemotherapy-related bone marrow suppression in the context of a four-drug treatment regimen administered to patients who had received a median of four prior lines of therapy^22^. Importantly, no patients were withdrawn because of TRAEs, and 40% of patients were able to receive maintenance therapy as part of the study, including one patient who had received 74 cycles at the time of data cut-off. Collectively, these data demonstrate that a reduced panobinostat dose (10 mg) can be effective and tolerated as part of a quadruplet regimen.

The reported patient response to pano-RVd is similar to outcomes reported with pano-Rd in the phase 2 study by Chari et al. (ORR 44% vs. 41%)^7^. However, the inclusion of bortezomib within the pano-RVd regimen resulted, as expected, in a higher rate of AEs, particularly neuropathy and thrombocytopenia. The two study populations were similar in terms of exposure to prior therapies, and percentage of patients refractory to bortezomib and lenalidomide at time of study entry. However, the studies had different approaches to dosing panobinostat^7^. In the pano-Rd study, panobinostat was dosed every other week, rather than the 2-weeks-on/1-week-off schedule in the current study. This alternative dosing schedule used by Chari et al. may have enabled patients to tolerate higher doses of study treatment.

Anticipation of overlapping toxicities, particularly bone marrow suppression, led to the dosing strategy in this study, in which both lenalidomide and bortezomib were dose reduced relative to standard dosing of these agents. In spite of this strategy, dose reduction of lenalidomide, bortezomib and dexamethasone was required in 15%, 30%, and 30% of patients, respectively, and at least one treatment delay was required in 90% of patients. However, these reductions in dose enabled longer-term administration of all four agents, which presumably contributed to the sustained responses observed in some patients.

The small sample size is an important limitation of this study and inherent to phase 1 studies in general. While patients had received a substantial number of prior therapies, they were also relatively young, and thus it will be important to ascertain whether this quadruplet regimen could be tolerated in elderly patients^23^. However, the similar rates of TRAEs observed in patients <65 and ≥65 years old suggest that, indeed, pano-RVd may be tolerated in fit elderly patients.

This study was not designed or powered to definitively evaluate efficacy. However, the exploratory data reported herein are promising. Of note, in a previous study, pano-RVd was shown to have a favorable safety and efficacy profile in the front-line setting^24^. Panobinostat has also been shown to be effective and well tolerated when administered 1-week-on/1-week-off in a 28-day cycle in combination with lenalidomide and dexamethasone^7^ or with carfilzomib^25^, and once-weekly administration of bortezomib has been shown to improve the safety profile while maintaining efficacy^26–28^. As such, it would be of interest to evaluate a modified dosing regimen of pano-RVd using a 28-day cycle in which panobinostat is administered 1-week-on/1-week-off, along with weekly bortezomib, as such an approach may reduce the rates of thrombocytopenia and PN, while maintaining efficacy, and so translate efficiently into real-world practice^23^.

The relative lack of significant patient exposure to more recently approved agents such as carfilzomib, daratumumab, and elotuzumab is also a potential limitation of the study. Nonetheless, the results highlight the ability of panobinostat to re-sensitize patients to agents which, in most instances, they had previously become refractory to and were resistant. These data underscore the utility of panobinostat as an oral therapy with a unique, multifaceted mechanism of action that can partner with other agents to overcome resistance and enhance treatment response. Given the use of continuous/treat-to-progression therapy as a standard of care in MM, patients are more likely to become refractory to multiple agents after fewer lines of therapy, pointing to the need for additional treatment options for RRMM. As the dose and schedule of panobinostat are optimized, regimens incorporating this agent, such as pano-RVd, may contribute to further improvements in patient outcomes by targeting patterns of resistance to IMiDs and/or PIs, as well as to other novel agents^29^.

## Supplementary information

Supplementary Figures

Supplementary Table 1

Supplementary Table 2

Reproducibility checklist

## Data Availability

All data requests should be submitted to the corresponding author for consideration. Access to anonymized data may be granted following review.
