# Supplementary material for: Phase 1 open-label study of panobinostat, lenalidomide, bortezomib + dexamethasone in relapsed and relapsed/refractory multiple myeloma
Source: Blood Cancer J. 2021 Feb 5;11(2):20. doi: 10.1038/s41408-021-00407-5 (PMC7873303; doi:10.1038/s41408-021-00407-5)
Supplement: Supplementary file 1 — Supplementary Figures [file 41408_2021_407_MOESM1_ESM.docx]

**Phase 1 open-label study of panobinostat, lenalidomide, bortezomib + dexamethasone in relapsed and relapsed/refractory multiple myeloma**

**Supplementary figures**

**Supplementary Fig. 1** Dose-escalation scheme


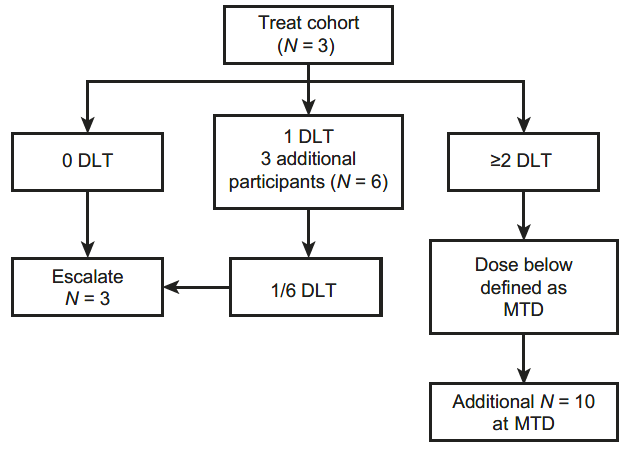


*DLT* dose limiting toxicity, *MTD* maximum tolerated dose.

**Supplementary Fig. 2** CONSORT diagram


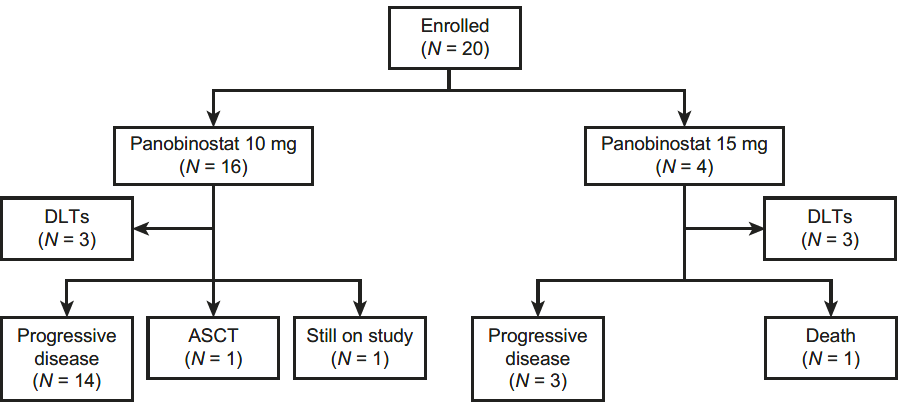


*ASCT* autologous stem cell transplantation, *DLT* dose-limiting toxicity
